# Prevalence and surgical outcomes of stage 3 and 4 pelvic organs prolapse in Jimma university medical center, south west Ethiopia

**DOI:** 10.1186/s12905-022-01992-8

**Published:** 2022-10-07

**Authors:** Demisew Amenu Sori, Stephan Bretones, Georges Mellier, Bertrand de Rochambeau

**Affiliations:** 1 Jimma University Institute of Health, Department of Obstetrics and Gynecology, Jimma University, Jimma, Ethiopia; 2 Urogynecology and Reconstructive Pelvic Surgeon, Saint Joseph-Saint Luc Hospital, Lyon, France; 3 Department of Obstetrics and Gynecology, Lyon 1 University, Villeurbanne, France

**Keywords:** Pelvic organ prolapse, Colposuspension, Surgical outcome, Simplified pelvic organ prolapse quantification (S-POPQ)

## Abstract

**Background:**

Pelvic organ prolapse (POP) affects about half of the women and affects their quality of life. The current study is, therefore, aimed at determining the prevalence and surgical outcomes of severe stage POP at Jimma University medical center from November 2016 to May 2018.

**Method:**

A Hospital-based cross-sectional study was conducted on all patients with stage 3 and 4 POP, who were admitted, and had surgery. Data were collected from the patient’s chart, and logbooks, which were filled up from entry till her discharge. A Simplified POPQ(S-POPQ) was used to stage the prolapse at admission, at discharge, and three months follow-ups.

**Results:**

Among 92 patients who were analyzed, POP accounts for 10.6% of all gynecologic admissions, and 43.8% of all gynecologic surgeries. The mean age of patients is 46 (± 12) years, and nearly 34% of the patients had stage 3 and 66% had stage 4 POP. Based on the type of prolapse, 93.5% of patients had stage 3 and more anterior vaginal wall prolapse (AVWP) and apical prolapse, while 57.6% had stage 3 or more posterior vaginal wall prolapse.

Out of 72 patients who had anterior colporrhaphy, 58.7% had anterior colporrhaphy with colposuspension. Out of 83 patients who had apical suspension, 48.2%, 39.8%, and 12% had uterosacral, sacrospinous, and Richardson respectively.

Ninety-seven patients had stage 0 or 1 POP at discharge while 90% of 20 patients who returned for follow-up at three months had stage 0 or 1 POP. Eight patients had surgery-related complications; bladder injury, urinary retention, Hemorrhage during SSLF, and rectal injury.

**Conclusion:**

The prevalence of pelvic organ prolapse is high and the majority of patients presented with advanced-stage pelvic organ prolapse, with a long duration of symptoms and associated problems. The surgical techniques used have resulted in a high immediate success rate of 97% and 90% at discharge and three months follow up respectively. Therefore, awareness creation activities are important to facilitate an early presentation for treatment to improve the quality of life and the current surgical technique; native tissue vaginal repair (NTVR), being practiced in the setup has had better success.

## Background

Pelvic organ prolapse (POP) comprises the descent of one or more aspects of the vagina and uterus [1]. The prevalence of symptomatic POP was much lower (3–6%) than the prevalence identified by examination (41–50%) in developed countries [1] and 6.3% and 55% respectively in Ethiopia [2] which is similar to the rural Gambia, West Africa [3]. Moreover, the global prevalence rate of POP is as high as 50% [4]. Advanced Anterior Vaginal Wall Prolapse (AVWP) is found to be correlated strongly with apical prolapse and anterior vaginal wall defects that are surgically repaired usually require a concomitant repair of the apex [5]. POP accounted for nearly 41% of major gynecologic operations in Jimma University specialized hospital and AVWP was the major finding among others [6].

The etiologies of pelvic organ prolapse are multifactorial, which include childbirth-related with: multi-parity, older age, instrumental delivery [7]; overweight or obesity [8], diabetes, causes of increased intra-abdominal pressure like COPD, hypertension, and collagen weakness [9, 10].

Anterior colporrhaphy is the commonly used traditional treatment modality for anterior vaginal wall prolapse with high recurrence rates of prolapses leading to reoperation or use of mesh in some setups. A national survey on the management of anterior vaginal prolapse in South Africa showed that anterior colporrhaphy was done by 85.5%, vaginal paravaginal repair by 41.9%, and mesh was used by 55.1% of the respondents [11].

A randomized controlled trial comparing anatomical and functional outcome between vaginal colposuspension and transvaginal mesh in France, have shown that the native-tissue colposuspension technique used in their series showed better anatomical success than previous reports of traditional procedures, and this calls for the necessity to spread and standardize colporrhaphy techniques, and in particular, vaginal colposuspension, which is effective, safe and has a lower complication rate [12].

Treatment success of POP varied from 19.2 to 97.2% depending on the definitions used. Definitions of success using all anatomic support to be proximal to the hymen had the lowest treatment success (19.2–57.6%), while the definition of the absence of prolapse beyond the hymen had a treatment success of 94%. Furthermore, subjective cure (absence of bulge symptoms) occurred in 92.1%, and subjective cure was associated with significant improvements in the patient’s assessment of both treatment success and overall improvement two years after surgery [13].

POP commonly affects parous women and its treatment depends on the severity of the prolapse, its symptoms, the woman’s general health, and surgeons’ preference and capabilities. Options available for treatment are conservative or mechanical for women with a mild degree of prolapse, those who wish to have more children, and those women unwilling to undergo surgery, or surgical interventions for others [14–16].

Treatment of POP varies from country to country and center to center with varying outcomes. Even though the current state of pelvic organ prolapse reconstructive surgery considers graft or mesh than vaginal native tissue repair (VNTR) for better long-term outcomes of AVWP, which has the highest recurrence rate, there are concerns with the use of mesh. A study showed treatment of anterior vaginal wall prolapse has a significant improvement of the point Ba in the polypropylene mesh group compared to the traditional anterior colporrhaphy, however prepubic hematoma and exposition of the mesh were the complications observed. There is no difference in vaginal symptoms and quality of life on follow-up [17].

In another study to compare anterior colporrhaphy and mesh at three years of follow-up, mesh reinforcement significantly reduced anatomic recurrences of anterior vaginal prolapse, but no difference in symptomatic recurrence was noted and the mesh erosion rate was found to be as high as 19% [18]. Furthermore, in a study to review the long-term results of an intraoperative decision to repair or not to repair associated vault and posterior compartment defects after a total hysterectomy and anterior vaginal wall suspension for uterine and bladder prolapses, a group with anterior vaginal wall suspension for uterine and bladder prolapses had low prolapse recurrence, additional repair, and higher success rate at 3 years follow-up [19, 20].

On the other hand, a study conducted to analyze the long-term outcomes of severe pelvic organ prolapse treated by VNTR showed that it is effective, safe, durable, improved POP-related symptoms, and sexual function [21]. Moreover, a retrospective review of pelvic organ prolapse recurrence after Anterior Vaginal Wall Suspension (AVWS) procedure for stage 2 anterior compartment prolapses showed that there was a low anterior compartment reoperation rate, minimal morbidity, and a low rate of secondary Stress Urinary Incontinence, though about a third required prolapse repair in different compartments [22]. A study on hysteropexy-based surgery compared with a hysterectomy and or other concomitant reconstructive surgery for pelvic organ prolapse has shown that hysteropexy is highly associated with reoperation rate compared with hysterectomy [23].

Furthermore, there are other treatment approaches for POP including, laparotomy, laparoscopy, and robot‐assisted laparoscopy. A systematic review on the Role of Laparoscopic Surgery in the Treatment of Advanced Uterine Prolapse showed that minimally invasive surgery can be used efficiently as an alternative to open surgery in the treatment of severe uterine prolapse [24]. Likewise, a 2 years prospective double center study on Laparoscopic Lateral Suspension (LLS) with mesh for apical and anterior Pelvic Organ Prolapse showed that at 2 years 89% of patients were asymptomatic, the anatomic success rate was 94.2% for the anterior compartment, and 94.9% for the apical compartment. This study concluded that LLS for the treatment of apical and anterior POP is a technique with optimal results in terms of safety and effectiveness after 2 years of follow‐up [25].

Even though the surgery for POP in Ethiopia is expected to vary from center to center, evidence is scarce on the surgical techniques and outcomes in Ethiopia in General and Jimma University Medical Center in particular. Thus, it is important to describe the surgical technique and the immediate outcomes of surgery. The present study is, therefore, to determine the prevalence and surgical outcomes of POP in Jimma university medical center from Nov 2016 to Feb 2018.

## Methods

A Hospital-based cross-sectional study design was conducted on all patients with POP using a retrospective review of their charts, who were admitted to Jimma University Medical Center (JUMC) Gynecology ward, had surgery from Nov 2016 to May 2018, and who meet the inclusion criteria. English version pretested data collection format was used to collect Data on Sociodemographic, obstetric variables, and physical examination findings by trained gynecology and obstetrics residents and the surgeons from a patient chart, operation logbook, and discharge logbook which were filled up from the entry of the patient to the hospital till her surgery, the postoperative period until discharge. The POP is staged using the simplified pelvic organ prolapse quantification (S-POPQ) system.

All patients with POP were followed up daily till discharge. At discharge, a pelvic examination was done to determine the outcome of the surgery as almost all women will not comply with the three months follow-up if there is no problem. In this study, the outcome variables were surgery-related complications, stage of the POP at discharge, and three months. The cure rate for prolapse surgery was defined at Simplified-POPQ ≤ 1 at discharge and three months of follow-up[26].

The Collected data were entered into Epidata version 3.1, cleaned, and analyzed using SPSS version 20, and interpretation, discussion, and recommendations were made based on the findings.

An official letter was obtained from the Institutional Review Board of Jimma University to conduct this research and get permission from the Hospital. After permission was obtained, data were collected from patients' chart, operation logbook, and from discharge logbook which was filled up during the study period. The outcome of this study has been communicated to the Department of Obstetrics and Gynecology and the Hospital.

The technique of colposuspension in this study (the new technique) is described in [12, 27]: Surgery begins with medial colpotomy along the vagina from apex to 15 mm from the urethral meatus. The bladder is separated from the pubovaginal fascia, leaving the fascia adhering to the vaginal tunica. Dissection extends laterally under the base of the bladder, penetrating deep into Retzius’ space, cutting the superior perineal aponeurosis.

A non-resorbable suture (Ethibon no. 1 or prolene no. 0) is anchored to the internal side of the vagina on the pubovesical fascia. The entire fascia is sutured, from the bladder neck to the vaginal apex, where a deep suture includes the bladder pillar fibers. Colposuspension is bilateral, suspending the entire anterior vaginal wall. Away from the colposuspension suture, the vaginal flap is doubled by cleaving between the pubovesical fascia within and vaginal epithelium without on each side. A 2-cm skin incision is made in the inguinal fold at the pubic spine. The anterosuperior pubic spine and inguinal ligament insertion are visible. A suture passer is introduced into Retzius’ space, meeting the surgeon’s finger in the dissection space. The colposuspension suture and needle are brought up to the abdominal incision. The same procedure is performed on both sides. Left and right pubovesical fascias are sutured, overlapping, onto one another, economic vaginal resection is performed, and the anterior colpotomy is closed with a running suture from the urethral meatus to the vaginal apex. Colposuspension sutures are tied to the fibrous structures against the anterosuperior pubic spines: i.e., on the inguinal ligament insertion, forming a solid anchorage. The sutures are tightened without tension, rendering the anterior vaginal wall horizontal, and suspended at both the bladder neck and vaginal apex.

### Operational definition

For the simplified POPQ (S-POP), the four areas examined included the anterior and posterior vaginal walls, the apex, and the cervix. If a subject was status post-hysterectomy, then only three measurements were taken: the anterior and posterior vaginal walls and the cuff scar/apex. No measuring devices were required for the S-POP, and the investigators had to use estimates for identifying those points on the anterior and posterior vaginal segments that were used to represent the respective walls [26].

## Result

140 patients had surgery for POP among the total of 1326 gynecologic admissions and among the total of 320 gynecologic elective surgeries from November 1, 2016, to March 31, 2018, in Jimma University Teaching Hospital. Among the total of 140 patients with POP who had surgical treatment, 48 were excluded as their data was incomplete; and data of the remaining 92 were analyzed. The prevalence of POP is 10.6% of all gynecologic admissions and 43.8% of all gynecologic surgeries.

The age of the patients ranged from 21 to 70 years with a mean of 46 (± 12) years and 49 (53.3%) were younger than 49 years. Sixty-five patients (70.7%) were living in rural areas and 8 (8.7%) of them were having one or no child. Sixty-five percent of patients were grand multiparous, 3 were para I, and one was nulliparous. Sixty-six percent of patients were married and 18 (19.6%) were widowed. Seventy-three (79.3%) of patients had symptoms of POP for five or fewer years with a median duration of 2.8 years while 6 patients were symptomatic for more than ten years. Fifty-six patients (60.9%) were sexually active and three had associated urinary incontinence. Among the reasons for not being sexually active was an absence of sexual interest due to bulge among 16 (44.4%) patients and associated urinary incontinence among 3 patients (Table 1).

**Table 1:** Socio-demographic characteristics and obstetric variables of anterior vaginal wall prolapse in JUMC from Nov 1, 2016, to March 31, 2018

Variable		Number (N = 92)	Percent
Age in years	20–29	7	7.6
	30–39	19	20.7
	40–49	23	25.0
	50–59	23	25.0
	≥ 60	20	21.7
Parity (current)	≤ I	4	4.3
	II-IV	28	30.4
	≥ V	60	65.2
Marital status^*^ (N = 87)	Single	7	7.6
	Currently married	61	66.3
	Widowed	18	19.6
	Divorced /separated	1	1.1
Residence	Urban	27	29.3
	Rural	65	70.7
Number of living children	≤ 1	8	8.7
	2–4	38	41.3
	≥ 5	46	50.0
Duration of AVWP with symptoms in years (median = 2.8 years)	≤ 5	73	79.3
	6–10	13	14.1
	> 10	6	6.5
Do you have urinary incontinence?	Yes (SUI)	3	3.3
	No	89	96.7
Are you sexually active? (N = 56)	Yes	56	60.9
	No	36	39.1
Reasons for not sexually active (N = 36)	No partner	17	47.2
	No interest	16	44.4
	Urinary incontinence /bulge	3	8.3

Asterisk shows the different number of study subjects for the variables

All patients with POP gave birth vaginally and 14 (15.2%) had chronic medical illnesses. Fifty-five percent of patients were in menopause when they develop POP and 18 (19.6%) were less than six years in menopause and the rest were six years and more in menopause. Four patients had previous surgery for POP (Table 2).

**Table 2:** Risk factors of anterior vaginal wall prolapse (AVWP) in JUMC from Nov 1, 2016, to March 31, 2018

Risk factors of AVWP		Number (N = 92)	Percent
Mode of delivery	Vaginal	92	100.0
Chronic medical illnesses (N = 14)	Yes	14	15.2
	No	78	84.8
Type of Chronic medical illnesses	Asthma	1	1.1
	Diabetes	2	2.2
	Hypertension	10	10.9
	HIV/AIDS	1	1.1
Is the woman Menopause when she develops AVWP?	Yes	51	55.4
	No	41	44.6
Duration in menopause when in years (range = 1–20)	≤ 5	18	19.6
	6–10	15	16.3
	> 10	18	19.6
Previous surgery was done (hysterectomy and or surgery for POP)	Yes	4	4.3
	No	88	95.7
Type of previous surgery^*^	SSLF without hysterectomy (Richardson)	1	25.0
	VH + AC + PC	1	25.0
	VH + AC	2	50.0

^*^^VH= vaginal hysterectomy, AC= anterior colporrhaphy, PC= posterior colporrhaphy^

**Fig. 1:**
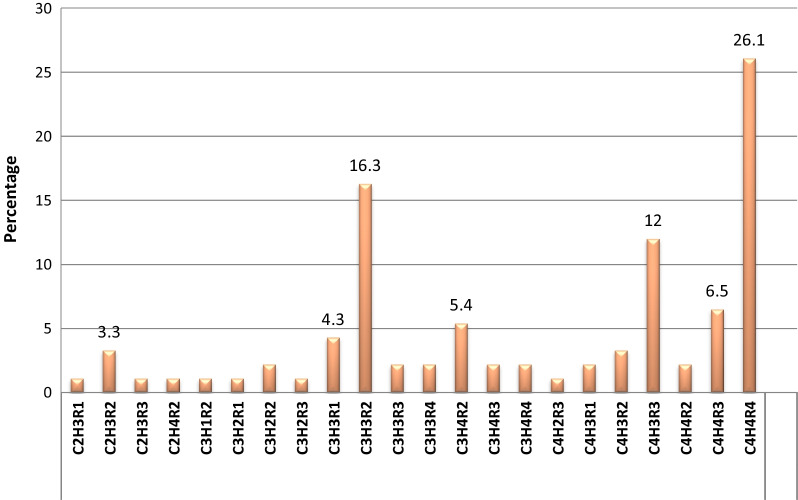
The combined stage of POP before surgery in JUMC from November 1, 2016, to March 31, 2018. CHR: C = Anterior vaginal wall prolapse (AVWP), H = Apical prolapse, R = Posterior vaginal wall prolapse (PVWP)

Nearly 94% of patients had stage 3 and 4 anterior vaginal walls prolapse (AVWP) and apical prolapse based on Simplified Pelvic organ prolapse quantification (S-POPQ). Three patients had vault prolapse following previous surgery for POP. The majority, 53 (57 0.6%) of patients with posterior vaginal wall prolapse (PVWP) had stage 3 and above POP. Sixty-six percent of patients had stage 4 POP as a final stage. Twenty-six percent of patients had combined stage 4 (C_4_H_4_R_4_) POP while 16.3% had combined stage C_3_H_3_R_2_ (Fig. 1).

Regarding the management of POP, 72 (78%) of patients with POP had anterior colporrhaphy and 54 (58.7%) had anterior colporrhaphy with colposuspension. Ninety percent of patients had apical treatment for their prolapse, which includes uterosacral ligament suspension among 40 (43.5%), sacrospinous ligament fixation (SSLF) among 33 (34.8%), and SSLF without hysterectomy (Richardson) among 9 (9.8%). Nearly 85% of patients had posterior colporrhaphy, and of these 7.6% had additional anterior rectal plication. Eighty-seven percent of patients had perineorrhaphy, and 9 (9.8%) had colpohysterectomy (Roulhier).

Spinal anesthesia was used in 89 (96.7%) cases and the duration of surgery ranged from 50 to 210 min with a mean duration of 114 (± 36) minutes. Nearly 84% of patients had bladder drainage for 48 h or less (Table 3).

**Table 3:** Stages of pelvic organ prolapses using Simplified Pelvic Organ Prolapse Quantification (S-POPQ) and its treatment in JUMC from Nov 1, 2016, to March 31, 2018

Variables		Number (N = 92)	Percent
Anterior vaginal wall prolapse	Stage 2	6	6.5
	Stage 3	37	40.2
	Stage 4	49	53.3
Apical prolapse	Stage 1	1	1.1
	Stage 2	5	5.4
	Stage 3	44	47.8
	Stage 4	42	45.7
Posterior vaginal wall prolapse	Stage 1	8	8.7
	Stage 2	31	33.7
	Stage 3	25	27.2
	Stage 4	28	30.4
The final stage of the prolapse	Stage 3	31	33.7
	Stage 4	61	66.3
Type of pelvic organ prolapse surgery	Anterior colporrhaphy	72	78.3
	Anterior colporrhaphy with colposuspension	54	58.7
	Vaginal apical treatment	83	90.2
	Posterior colporrhaphy	78	84.8
	Rectal anterior plication	7	7.6
	Perineorrhaphy	80	87
	Colpohysterectomy with posterior myorrhaphy (Roulhier)	9	9.8
Type of vaginal apical treatment (N = 82)	SSLF without hysterectomy (Richardson)	10	12
	SSLF (Richter)	33	39.8
	Uterosacral ligament	40	48.2
Type of anesthesia	Spinal	89	96.7
	General	3	3.3
Duration of surgery in minutes	= < 60	7	7.6
	61–120	51	55.4
	121–180	32	34.8
	> 180	2	2.2
Duration of postoperative bladder catheterization in hours	≤ 48	77	83.7
	> 48	15	16.3

There were 8 (8.7%) surgery-related complications; among these were bladder injury, rectal injury, urinary retention, and hemorrhage during SSLF seen in 4, 1, 3, and 3 patients respectively. All patients who had surgery for any of POP, had their prolapse staged at discharge. Accordingly, 72 (78.3%), 16 (17.4%), and 4 (4.3%) had stage 0, 1 and 2 respectively at discharge. Out of 20 patients who had returned at 3 months for follow up 16 (80%) had stage 0, 2 patients had stage 1, and another 2 patients had stage 2 POP (Table 4). None of the patients had the preoperative symptoms both at discharge and three months follow-up.

**Table 4:** Surgical outcomes of pelvic organ prolapses managed in JUMC from Nov 1, 2016, to March 31, 2018

Variables		Number (N = 92)	Percent
Surgery related complications	Yes	8	8.7
	No	84	91.3
Types of complications (the same patient can have more complications) [8]	Bladder injury	4	50
	Rectal injury	1	12.5
	Urinary retention	3	37.5
	Hemorrhage during SSLF	3	37.5
Stage of AVWP at discharge	Stage 0	72	78.26
	Stage 1	16	17.39
	Stage 2	4	4.35
Stage of apical prolapse at discharge	Stage 0	81	88.04
	Stage 1	11	11.96
Stage of PVWP at discharge	Stage 0	92	100.0
The final stage of the prolapse at discharge	Stage 0	72	78.26
	Stage 1	16	17.39
	Stage 2	4	4.35
Symptoms at discharge	No	92	100.0
Follow up visit at 3 months	Yes	20	78.26
	No	72	70.3
Stage of AVWP (N = 20)	≤ Stage 0	18	90.0
	Stage 1	2	10.0
Stage of apical prolapse(N = 20)	≤ stage 0	16	80.0
	Stage 1	4	20.0
Stage of PVWP	≤ stage 0	20	100.0
The final stage of POP	stage 0	16	80.0
	Stage 1	2	10.0
	Stage 2	2	10.0
Symptoms at 3 months [20]	No	20	100.0

## Discussion

Pelvic organ prolapse is a common problem globally and its prevalence varies from country to country and set up to set up based on whether the diagnosis was made based on symptoms or physical examination [1]. The 10.6% prevalence of POP in this study is higher than the 1.4% study done in Nigerian Hospitals [28, 29] and similar to the study done in Kilimanjaro, Tanzania which ranges from 6.1 to 12.9% based on the stage of the prolapse [30]. This difference might be from the difference in socio-demographic characteristics of patients and modes of delivery as in our case all patients with POP had delivered vaginally. On the other hand, the prevalence of POP among elective gynecologic surgeries is more or less similar to the previous study in the same setup (40.7 vs. 43.8%) [6]. This shows that pelvic organ prolapse remained a major gynecologic problem in this setup.

More than half of the patients in our study are younger than 49 years and nearly 71% were residing in rural areas which are similar to a previous study in the hospital [6]. This finding can be explained by the lifestyle of women in the rural area where they are engaged in heavy work like fetching water and carrying for long distances, farming, and other related activities [31]. All patients gave birth vaginally and the majority of the patients (65%) in this study are grand multiparous which is said to be the major risk factor of POP and this is similar to the study in Nigeria [28]. A study in the USA has shown that a single vaginal birth has increased the odds of POP to 9.7 and there is no increase in odds of prolapse in subsequent deliveries [32], in another study, however, the prevalence of POP has increased with subsequent term vaginal deliveries [33]. Furthermore, Caesarean section is found to have a limited primary preventive effect on pelvic floor dysfunction at a population level [33]. This implies that even though the first vaginal delivery is the most important risk factor for the occurrence of POP, subsequent vaginal deliveries are also important risk factors.

The majority of patients had POP with symptoms for five or fewer years with a median duration of 2.8 years. This finding is similar to the study done in Nigeria [28]. Pelvic organ prolapse impacts the sexual life of women and similarly, 20.6% of patients with AVWP are not sexually active in our study because of lack or decreased libido and urinary incontinence. Another study has also shown that pelvic floor symptoms are significantly associated with reduced sexual arousal, infrequent orgasm, and dyspareunia [34, 35]. As a result, studies recommend that the impact of POP on quality of life (QoL) and sexual dysfunction should be part of the POP evaluation and therapeutic process using standard questionnaires [36, 37]. Urinary incontinence in this study is seen in only 3 patients (3.3%) and when compared to the prevalence in literature it is very low which could be explained by the advanced stage POP in our study while Stress urinary incontinence (SUI) is found to be higher among stage II POP patients (55%) than stage IV POP patients (33%) [38].

Although childbirth injury is the major risk factor for POP, there are many other contributing factors to POP [5, 13, 39]. Among these are chronic medical illnesses like Diabetes, Hypertension, Obesity, menopause and chronic obstructive lung diseases can be mentioned [9, 10]. In our study, 15.2% of patients had chronic medical illnesses and hypertension is the major one. This could be related to the neurovascular compromise related to this disease and also chronic illness-related malnutrition. Fifty-five percent of patients were in menopause when they develop POP and majorities were in menopause for more than five years (36% of total POPs).

In our study, all patients had some degree of all compartment prolapses and the majority of the patients had advanced-stage POP. Nearly 94% of patients had stage 3 and 4 AVWP and apical prolapse while 57.6% had PVWP based on Simplified Pelvic organ prolapse quantification (S-POPQ). This finding is similar to a previous study done in the same setup regarding the prevalence of AVWP where 99.4% of apical prolapse had a cystocele and 100% had stage 3–4 apical prolapse [6]. The difference is with the prevalence of PVWP where it is only reported in 16.3%. Furthermore, AVWP is found to be more highly associated with apical prolapse than PVWP [5, 30] as is also seen in our study. This correlation implies that anterior vaginal wall defects that are surgically repaired usually require a concomitant repair of the apex. The final stage of prolapse (all compartments) in this study is stage 3–4 and the final stage of prolapse is 4 in 60% of patients. This implies that patients with pelvic organ prolapse seek care late in this setup.

Out of Seventy-eight percent of patients with AVWP had anterior repair (anterior colporrhaphy), 58.7% had anterior repair with colposuspension using the technique described in the method part [12]. Stage 3 and above AVWP require additional procedures like the use of vaginal mesh or other anterior colposuspension procedures in addition to anterior colporrhaphy because of the high risk of recurrence which is found to be 27% to 42% after native tissue repair (anterior colporrhaphy) [17]. Use of transvaginal mesh in anterior vaginal wall prolapse repair is found to have good short-term to medium anatomic and subjective outcomes; however, its use results in higher rates of surgical complications (longer duration of surgery, higher rates of hemorrhage, bladder perforation, new stress urinary incontinence, and reoperation to correct mesh exposure) with no difference in reoperation rates for recurrent prolapse compared to anterior colporrhaphy [1, 4, 20, 40]. Furthermore, there was no difference in the vaginal symptoms (perception of prolapse) and quality of life (QoL) between the two groups [19]. A study has also shown that anterior vaginal wall suspension for symptomatic AVWP offers native tissue vaginal repair with minimal morbidity and low AVWP rate at intermediate to long-term follow-up [41]. Accordingly, in our study, 82.8% of patients with anterior colporrhaphy had additional anterior colposuspension. Thus, vaginal native tissue repair (VNTR) as a surgical treatment for severe POP was found at the long-term follow-up to have a subjective cure rate of 97.3% and the objective cure rate of 91.1%. This concluded that VNTR is effective, safe, and durable, and improves POP-related symptoms and sexual function [39]. Considering the current international warning about the health risk of the use of transvaginal mesh (class III = high-risk device) in general and the treatment cost and unavailability of mesh in low-resource countries like Ethiopia in particular, the use of this non-inferior VNTR is crucial.

The duration of surgery for pelvic organ prolapse depends on the type of POP repair, operator’s skill, and preoperative preparation of the surgical team. The whole duration of surgery to repair all types of POP in this study ranged from 50 to 210 min with a mean duration of 114 (± 36) minutes. This study is comparable with the previous studies [17, 40]. The duration of bladder drainage after anterior repair varies from center to center and is based on the type of procedure performed and related complications. In our study, nearly 84% of patients had bladder drainage for 48 h or less, and 4.3% and 3.2% had bladder injury and urinary retention during the surgery and postoperatively respectively. This is similar to the duration of bladder catheterization and higher for intraoperative and postoperative complications compared to a study conducted to assess the anterior colporrhaphy vs transvaginal mesh [40].

All patients who had POP surgery in our study, had their prolapses staged at discharge. Accordingly, 72 (78.3%), 16 (17.4%), and 4 (4.3%) had stages 0, 1, and 2 respectively, and has no preoperative symptoms at discharge. Out of 20 (21.7% of total) patients who returned at 3 months for follow up 18 (90%) had stage 0 or 1 POP while 2 had stage 2 POP and had no bulge symptoms. The success rates of surgery as defined by stage after repair of ≤ 1 using native tissue repair in this study is high compared to other studies [13, 18, 21, 42].

Limitation of the study: this analysis is based on retrospective data and some of the sociodemographic and obstetric variables were not assessed. In addition, the surgical outcome assessed only the immediate outcome at discharge and few patients at 3 months and was not possible to get patients for long-term follow-up.

## Conclusions

POP surgeries are the major elective gynecologic surgeries in the hospital, Vaginal delivery and grand multiparity are the possible major risk factors identified in this study, and POP has impacted the sexual life of women. Vaginal native tissue repair used for all POP in this study resulted in a high immediate success rate (97% and 90% at discharge and 3 months). There was no major Intra and postoperative complication encountered in all patients. JUMC has to work on advancing the care of women with POP by training more expertise in Urogynecology and reconstructive pelvic surgery and availing the necessary supplies and facilities. The government has to use different platforms to increase community awareness of the risk of grand multiparity and the use of family planning to control grand multiparity. The surgical technique used in this study might be replicated in other setups in Ethiopia as it has a high immediate success rate.

## Data Availability

The information supporting the conclusions of this article is included within the article. Further information can be obtained from the corresponding author upon request.

## Abbreviations

JUMC: Jimma university medical center
POP: Pelvic organ prolapse
AVWP: Anterior vaginal wall prolapse
PVWP: Posterior vaginal wall prolapse
VNTR: Vaginal native tissue repair
AWVS: Anterior vaginal wall suspension
